# Democracy, epistemic agency, and AI: political epistemology in times of artificial intelligence

**DOI:** 10.1007/s43681-022-00239-4

**Published:** 2022-11-22

**Authors:** Mark Coeckelbergh

**Affiliations:** Department of Philosophy, University of Vienna, Vienna, Austria

**Keywords:** Democracy, Political epistemology, Epistemic agency, Artificial intelligence, Epistemic bubbles, Fake news

## Abstract

Democratic theories assume that citizens have some form of political knowledge in order to vote for representatives or to directly engage in democratic deliberation and participation. However, apart from widespread attention to the phenomenon of fake news and misinformation, less attention has been paid to *how* they are supposed to acquire that knowledge in contexts shaped by artificial intelligence and related digital technologies. While this topic can also be approached from an empirical angle, this paper contributes to supporting concerns about AI and democracy by looking at the issue through the lens of political epistemology, in particular using the concept of epistemic agency. It argues that artificial intelligence (AI) endangers democracy since it risks to diminish the epistemic agency of citizens and thereby undermine the relevant kind of political agency in democracy. It shows that next to fake news and manipulation by means of AI analysis of big data, epistemic bubbles and the defaulting of statistical knowledge endanger the epistemic agency of citizens when they form and wish to revise their political beliefs. AI risks to undermine trust in one’s own epistemic capacities and hinder the exercise of those capacities. If we want to protect the knowledge basis of our democracies, we must address these problems in education and technology policy.

## Introduction: AI, democracy, and epistemic agency

The general concern that artificial intelligence (AI) technologies are a threat to a democratic society is well known in public discourse, as are phenomena such as epistemic bubbles and echo chambers. For example, a 2019 article in MIT Technology Review already warned that AI is a ‘threat’ to democracy [29] and recently The Telegraph reported that the BBC ‘wages war on online echo chambers’ [55]. But why, exactly, is AI a threat to democracy and why are these phenomena a problem? This paper brings political epistemology to bear on this discourse. In particular, it uses the concept of epistemic agency to support concerns about AI, knowledge, and democracy.

In order to refine the question of this paper, let us look at the central concepts: AI, democracy, and epistemic agency.

AI is a complex phenomenon and comprises a wide range of technologies and techniques. Here, I will focus on AI in the sense of machine learning algorithms, which are used in digital social media contexts and on the Internet. Machine learning is a method of data analysis that enables the system to identify statistical patterns in data with minimal human intervention. In social media contexts, it can be used to search content, make recommendations, recognize images or speech, profile users and target them with personalized advertising, analyze sentiments in text, or create new content. Consider for example recommender and search algorithms used by Facebook, Twitter, and Google, but also deepfakes: videos and other media generated by machine learning algorithms in which a person in an existing image or video is (partly) replaced with someone else’s likeness. For example, it is now possible to make a video of a politician and have him or her say things he never said.

Theories and definitions of democracy come in a wide variety and, therefore, also vary considerably on what is expected of citizens. Some representative versions are “thinner” and suffice with asking that citizens vote every 4 or 5 years, whereas others—let us call them “thicker” ones—require that citizens directly engage in democratic deliberation [27], Cohen [13]; [9, 21] or agonistic struggle [36]. The use of the words “thin” (or “weak”) and “thick” (or” strong”) have a history in political theory (see for example already [3]). For the purpose of this paper, I shall mean with “thin” conceptions of democracy those that refer to voting and representation, whereas “thick” conceptions require direct participation of citizens. There are also attempts to find understandings of democracy that do not neatly fit these categories. For example, recently Landemore [32] has proposed a nonelectoral version of political representation with a large body consisting of randomly selected citizens—thus going some way to capture the spirit of direct democracy while keeping representation.

In this paper, I am interested in the knowledge basis of democracy. All varieties assume politically relevant knowledge on the part of the citizens. For example, one can expect that voters inform themselves about the political programs of candidates. In addition, in a deliberative and participative democracy, citizens are supposed to have knowledge about the issues at hand and be able to exercise what Habermas called communicative rationality [27] or to engage in agonistic political struggle [36], contesting each other’s point of view. This raises not only questions about *what* people need to know in a democracy but also *how* people know (process, know-how, and skill). Political epistemology is especially interested in the latter set of questions. How do citizens acquire politically relevant knowledge, for example political beliefs? What makes it knowledge? In addition, what is the relation between truth and democracy? For example, recently there has been a so-called ‘epistemic turn’ in deliberative democracy theory, which is concerned about the role of truth in politics and democracy and which rejects agnosticism with regard to the truth value of political claims [31] while trying to avoid the rule of experts [42] as proposed by Plato. For example, Estlund [21] has argued for ‘epistemic proceduralism’ which rejects the Platonic model but inserts an epistemic dimension in democratic politics by emphasizing discussion and interpersonal reasoning [22].

In this paper, however, my question is not about truth and democracy as such but about *epistemic agency*: do citizens have sufficient epistemic agency in the light of AI?

The term epistemic agency has not been used very often in relation to digital technologies (an exception is [26], who wrote about the Internet and epistemic agency), but is a familiar term in epistemology. Epistemic agency concerns the question regarding control over one’s beliefs (Scholosser [49]) and how these beliefs are formed and revised. We have the capacity to reflect on our beliefs, but it is not clear how much control we have over them. We *wish* to have control over our beliefs, but do we? In epistemology there is a long-standing discussion about the voluntariness of belief formation [30] and its relation to normative concepts such as responsibility [51]. In social epistemology, there is also discussion about the influence of the social on belief formation: my beliefs are related to a wider knowledge community and there is something like “collective knowledge” (for some key papers in the field see for example [24]). Here, I focus on the formation of political beliefs by citizens and on politically relevant epistemic agency in the light of technologies.

Political democratic agency seems to rely on epistemic agency, in the sense that as a citizen in a democracy I need to have some control over the formation of my political knowledge. Reflection on one’s beliefs and willingness to discuss them publicly is especially important in deliberative and participative ideals of democracy, but others also assume that citizens have some control over their beliefs. If I am brainwashed by an authoritarian regime, for example, I lack such control and, therefore, lack also political agency with regard to my voting or my participation in democratic deliberations. In addition, if my political beliefs are manipulated, neither voting nor deliberative democracy seem to get off the ground: they all assume that citizens, whether as voters or as participants in deliberation, have control over their own political knowledge, for example over their political beliefs, which they then express by voting, argue for in a deliberative procedure, or defend in an agonistic struggle. Only non-democratic, authoritarian and totalitarian political orders do not require epistemic agency from their citizens because they are not supposed to have political agency other than supporting the regime. Forming one’s own political beliefs, reflecting on them, and discussing them with others is then even actively discouraged.

Recent developments in society and technology raise the question whether the epistemic basis of existing forms of democracy is still sufficiently strong—if it ever was. For example, a common complaint from intellectuals worried about the vulnerability of liberal democracies (and indeed an objection to democracy since Plato) is that citizens are insufficiently educated to vote in an informed way, let alone to participate in other democratic processes and procedures. In addition, even in the twenty-first century, there are still enough authoritarian regimes that aim to diminish the political and epistemic agency of their citizens, for example by distorting truth via control of the classical mass media (TV and radio), which are still surprisingly influential, especially outside the Western world. However, a relatively new phenomenon is the use of artificial intelligence (AI) and big data by governments and big tech companies, which has a pervasive influence on citizens via digital social media, and which, therefore, is also likely to impact the knowledge basis of democracy. This suggestion needs further investigation: what is the influence of AI on political knowledge and knowledge formation, and how does it function?

While epistemic agency has been under attack for a while (also in Western democracies, for example by sensationalist and one-sided reporting in newspapers and on TV) and while the basis of such concerns can also be studied from an empirical point of view, philosophers and political theorists can contribute, and have contributed, with arguments and conceptual work. For example, there has already been thinking about how to make technical knowledge developed by mathematicians relevant to democratic theory [25] and there has been work on phenomena such as fake news [15, 45], filter bubbles [43] and epistemic bubbles [38]. There is also an emerging body of work on AI and democracy in general (for example [46], Sudmann [52]; [37], O’Neil [39]). For example, Danahar [16] has warned for what he calls ‘algocracy’: a governance system ‘organized and structured on the basis of computer-programmed algorithms’ (p. 247). There is also more attention to political epistemology in general (for example Hannon and Ridder [28]). But while these efforts are relevant to the topic at hand (in particular, I will return to the phenomena of fake news and misinformation, epistemic bubbles, and statistical knowledge), more work is needed on the political-epistemic impact of technology, including AI. In this paper, I am interested in the relation between AI, knowledge, and democracy. Moreover, in line with much of the literature, I focus on the risks and dangers posed by AI. I fully acknowledge that AI might also have good effects on democracy (which is also under-studied), but this is not the topic of this paper. In addition, if we want “AI for democracy,” it is important to first understand the problems.

This paper asks these questions about the political epistemology of AI, with a focus on the influence of AI on the corrosion of *epistemic agency*. This enables to go beyond the usual claim that the mere existence of fake news and misinformation is the main epistemic problem for democracies, since it paints a broader picture of the epistemic problems raised by AI and stresses the impact on the *how* of political knowledge. It also complements existing literature in ethics of AI that tends to focus on opacity/transparency when it comes to the social-epistemic effects of AI (for example Burell [12]); to this discussion it brings an interesting new topic: epistemic agency. In particular, the paper zooms in on (1) trust in one’s own epistemic agency and (2) the influence of AI on the formation and revision of our beliefs—a topic which is currently not sufficiently addressed. While these claims could also be interpreted as empirical hypotheses, the emphasis of the paper is on *providing conceptual arguments and justifications* for why AI might be a problem for politically and democratically relevant epistemic agency.

First, I argue that fake news and misinformation is not just a problem at the level of *what* knowledge citizens need for democracy (for example, one could argue that democracy needs truth), but is especially damaging at the procedural, *how* level, since it destroys trust in the socio-epistemic environment and in the end in the one’s own epistemic capacities: when fake news is ubiquitous, I can no longer believe others but I also can no longer believe my own eyes, and perhaps I feel a kind “epistemic shame” when confronted with technologies that can fake anything and that outperform human capacities for deception and deception detection. This attacks my epistemic and, therefore, political democratic agency at a fundamental level, and opens the door to totalitarianism: AI knows my political beliefs, and it might even know them better than me, in the sense that it has knowledge about patterns in the data of my online behavior that I might not be aware of. This enables control by those who develop, use, and own AI, be it private companies or governments.

Second, I argue that belief formation and control over belief formation are also endangered by (intended) direct manipulation of beliefs by means of AI, for example AI-based micro-targeting as happened in the case of Cambridge Analytica, and by (often non-intended) phenomena such as epistemic bubbles and the defaulting of statistical knowledge over other types of scientific knowledge that rely on causal evidence. The latter phenomena present a risk to epistemic and political democratic agency not by offering fake news or by engaging in advertising-like forms of manipulation, but rather by creating an epistemic environment and knowledge architecture that re-enforces beliefs present in a particular community and that makes it less likely that one’s beliefs are confronted with scientific evidence, which renders the kind of belief formation and revision needed for democracy less likely and more difficult. Again this is an assault on citizens’ epistemic agency and, therefore, on political agency in a democracy.

Third, the paper ends with brief policy recommendations based on this analysis: if we care about maintaining or creating democracy, then regulation of technology use and development, next to reform of education that takes into account these phenomena, is mandatory. It points to some relevant existing and ongoing work in that area.

## Why fake news and misinformation are *extra* problematic for democracy

The phenomena of fake news and misinformation are by now well known (see for example Zimdars and McLeod [56]). Often it is used for political purposes. A well-known example is the so-called *Pizzagate*: in 2016, members of an Internet message board known for the circulation of extreme beliefs and conspiracy theories spread the fake news that Bill and Hillary Clinton used a pizza restaurant as a front for a podophile sex ring. This fake news then spread to Twitter, Reddit, and Facebook. The debunked story had real-world consequences such as death threats. In 2020, it made a comeback on Instagram and TikTok and was promoted by QAnon.^1^

There is widespread agreement that misinformation and fake news are a threat to democracy: they influence opinions and voting, lead to confusion about what is true and real, and undermine the epistemic quality of deliberation needed for deliberative democracy. For example, McKay and Tenove [34] have argued that online disinformation undermines a polity’s capacity to engage in communication characterized by the use of facts. While next to truth there may also be other epistemically relevant bases for democracy such as inclusion and emotions ([23], p. 7, one could also draw more directly on the works of Mouffe and Arendt), let us assume—in line with deliberative theories of democracy and with the mentioned epistemic turn in democracy theory–that truth is a necessary, though not sufficient condition for democracy.

The political importance of truth in democracy becomes especially clear when we consider totalitarianism. In her writings about the origins of totalitarianism, Hannah Arendt already pointed out that it is a feature of totalitarian regimes to distort the truth. In *The Origins of Totalitarianism* [2], she says that such regimes and movements have an ‘unsurpassed capacity to establish and safeguard the fictitious world through consistent lying’ (p. 499). Truth is the enemy of authoritarian and especially totalitarian regimes, which aim to control the epistemic environment in a way that support the establishment and maintenance of their power. To the extent that the use of AI enables the distortion of the truth, for example by means of changing search engines or the creation of fake videos (deepfakes) which are then spread via digital social media or by means of the intended and targeted spread of misinformation via social media and based on analysis of big data, it supports existing authoritarian and totalitarian regimes. For example, China has been accused of using search algorithms to make articles on Xinjiang and the Uyghur minority that are published by state media dominant in the search results,^2^ and it has been claimed that Myanmar authorities have repeatedly shared fake images and challenged the reality of evidence of human rights violations.^3^

But liberal democracies are also at risk. While currently citizens might still be confident in their abilities to identify misinformation (Barthel, Mitchell, and Holcomb [4]) and so far, the political impact of deepfakes has been limited (Langguth et al. [33]), AI is already creating confusion and this is likely to get worse when AI’s capabilities to produce fake news improve. It becomes increasingly difficult to distinguish deepfake videos from real ones. Manipulation of images was already possible before, but now, with AI, it is easy to make such videos and hard to recognize that they are fake. Moreover, propelled by social media, all this misinformation travels much faster and broader than ever before. Fake news in traditional communities—say rumors in a village that were false—had mainly local effects. Now the reach is global. In addition, while TV and newspapers were already mass media, there were still some editorial barriers, including fact checking. Now with the help of AI, everyone can easily generate new content and content that has many views is pushed by social media and spread all over the world, regardless of content and truth.

An example of a politically relevant deep fake was a 2019 digitally altered video that showed Nancy Pelosi, the speaker of the US House of Representatives, stammer drunkenly through a speech at a news conference. While the video was soon debunked, it was shared on Facebook and afterwards posted on Twitter by Donald Trump (then president), receiving millions of views.^4^

If and in so far AI creates these phenomena, then this is an epistemic agency-based argument for why it is a problem for democracy: in an environment where it is no longer clear what is true or not, real or not, I cannot exercise my capacities for epistemic agency. My epistemic environment is too distorted, and ultimately this also distorts my social and political environment: I will no longer trust. For example, Ovadya [40] has warned for an ‘infocaplypse’ and argued that technology is disrupting reality and, therefore, the accountability of representative government.

However, the problem with fake news and misinformation is not just that there are particular cases of lying and truth distortion, which destroys trust in others and, therefore, in society and in democratic politics. The problem is also that citizens can no longer believe their own eyes and hence start doubting and mistrust not only others but also *their own* epistemic capacities. If AI fakes increasingly more “believable,” then I start questioning my own capacities as an epistemic agent to distinguish truth from falsehood. In addition, there is a basis for this mistrust: if AI fakes the news (or might fake the news: the point is that I never know if AI was used or not), then I have effectively less epistemic agency: I have less control over the formation of my knowledge. Through the technology, others, in particular those who used AI or might use AI (again, it is the risk that creates the mistrust), are put in control of political knowledge and of *my* belief formation. This undermines my democratic political agency. Moreover, in analogy with what Günter Anders called “Promethean shame”—the realization that machines are much more perfect than we with our mortal and imperfect bodies, that they have a ‘humiliatingly high quality’ [1], (p. 30)—I might feel ashamed in the face of AI which has much greater epistemic control capacities than me. If I am deceived by another human, in politics or elsewhere, at least I have the same capacities to do so and may be able, in principle, to see the deception. If AI is so much better in faking (and in detecting fakes!), however, I feel disempowered in the light of those enormous deceiving capacities, which can no longer be countered by my human capacities for deception and deception detection. My epistemic agency in this respect is minimal compared to what AI can do.

Of course this ‘epistemic shame’ is in some ways misleading, in the sense that as a citizen I might be concerned about “AI” and its capacities, whereas in reality it is always AI-as-used-by-other-people, who have an interest in deceiving me. On the one hand, this is good news, because *if* we become aware of this and if this is about others exercising power over me, I can try to reveal this and try to take back the power. On the other hand, it is also bad news, since it means that there are people who purposefully but covertly attack the epistemic agency of other people, with many of them unaware that this is going on. AI thus gives some people (and their organizations) more power, which renders changing the power balance more difficult. Moreover, people who use AI against me will tend to have more knowledge about AI than me, and will be able to use that knowledge against me or at least in their own interests.

This leads us to the threat of technocracy. For a democracy, this loss of relative epistemic agency on the part of (most) citizens is detrimental and opens the door to technocracy and totalitarianism. If humans can no longer know what is fake or not, we need machines for that, since they know better than us what is fake and what is not fake. Or at least we need those humans—experts and technocratic politicians—who use the AI. But this also means we outsource both epistemic agency and political control to these machines, their expert users, and those who control the machines and the experts. This at least supports technocracy and potentially also totalitarianism. Totalitarian regimes have a new tool for the widespread distortion of the truth in their hands, one that is even more effective than political advertisement since it does not only influence citizens’ political beliefs as such, but also undermines (the belief in) their own epistemic capacities and agency. If we can no longer believe in ourselves as knowledge agents, then we have to throw ourselves in the hands of those who claim to know, for example because they have the right kind of tools that can extract patterns from large data. Using AI as a powerful political tool, large corporations and governments can thus monopolize the epistemic space and, therefore, the political space. The AI experts and the people who have power over them can claim to be Platonic philosopher-kings (or rather: data science kings) who have the knowledge to govern us. When ultimately only AI knows the truth, then those who control AI also control the truth and thereby the citizens. Once the epistemic agency of citizens is eroded in this manner, there is no longer a ground for democracy. As Plato may agree: why give power to citizens when they cannot distinguish between truth and falsehood? The AI barons and data emperors know better; they take and get the power.

One may object and point out that this presupposes that AI creates centralized, rather than decentralized knowledge and power. One could argue for a decentralized version of democracy supported by AI, or hope that AI may lead to a more efficient, market-based organization of the economic realm [35]. However, there are reasons to be pessimistic about these routes: like with the Internet, of which people also had high expectations in the direction of decentralization, currently such tendencies are hard to spot. Rather, as the Cambridge Analytica case and the mentioned example from China show, but also considering the use of AI in the context of the gigantic economic power and influence of Big Tech companies such as Google, Facebook, and Twitter, we see AI being used in ways that support oligopolist and authoritarian tendencies in Western countries and help authoritarian regimes.

## Beyond manipulation: non-intended effects of AI on belief formation and belief revision

Next to fake news and misinformation, there are of course all kinds of (other) forms of manipulation, in which AI may play a role. Consider the case of Cambridge Analytica [14], in which AI-based micro-targeting was used to manipulate the political opinions of people in elections. Data from Facebook users were used by the Trump presidential campaign in 2016 to target voters based on their personality types. AI was used to categorize voters but also to automatically test thousands of variations of an ad before deciding which one to place.^5^ Both uses of AI-enabled micro-targeting of voters. This is not a case of fake news, fake videos, or other means of disinformation, but nevertheless influences the formation of political beliefs—next to having other effects on democracy such as privacy violation (see for example [57]).

But there are also other ways in which AI and related technologies (especially social media) can influence citizens’ political beliefs, and in ways that are not necessarily intended. A well-known phenomenon in the recent literature is so-called epistemic bubbles (and earlier: filter bubbles [43]) and echo chambers. Like filter bubbles, the term ‘epistemic bubbles’ refers to a digital social media phenomenon in which exposure to information and arguments outside one’s own social media bubble is lacking and, therefore, other voices are excluded [38]. For example, if a person receives all their political news from Facebook and (almost) all their Facebook friends share these views, they are in an epistemic bubble. Filter bubbles and epistemic bubbles are created by means of recommender algorithms that select content that matches users’ profile and online history. Users can also deliberately block people with opposing views, the filtering can be intended or not. The creation of epistemic bubbles can also happen through search engines such as that of Google: search algorithms, which increasingly have a recommender dimension, create a bubble based on the users’ searches through personalization.

Just like misinformation, epistemic bubbles are not new. People living in small communities or closed organizations with like-minded people have often created such bubbles. This diminished their epistemic agency. The difference is that now, in an age and epistemic environment that is supposed to be shaped by modernity and the Enlightenment, the phenomenon of epistemic bubbles is reinforced and supported by technology. To some extent this already happened via older mass media such as TV. For example, one could argue that watching Fox News in the US puts you in a particular epistemic bubble. But now AI in combination with digital social media gives the phenomenon a fast and much broader impact and creates new and arguably more effective silos: search engines and recommender algorithms in digital social media analyze people’s data to create a bubble that fits their individual profile. This helps advertisers and Big Tech, but reduces perspectives and voices.

To the extent that the epistemic bubbles phenomenon is indeed happening (some studies seem to show that Internet users also find and engage with standpoints different from their own—for an overview of criticisms, see [54]), these bubbles and the resulting reduction of voices are intrinsically bad for democracy. This is so both according to a thin ideal of democracy (voters need choice and hence need to be exposed to different political voices) and especially according to a thick ideal: discussion in a deliberative and participatory democracy, for example, or agonistic struggle, are hardly possible if there is only one voice. For example, earlier it has been argued already that filter bubbles diminish information diversity and that discovering new perspectives is more difficult; this is a problem for deliberative democracy (Bozdag and van den Hoven [10], 252). In addition, Mouffe’s ideal of democracy as agonistic demands inclusion of voices. Epistemic bubbles work against that by reducing the number of voices. They also take away channels in which opposing viewpoints can clash, as Bozdag and van den Hoven argue for filter bubbles (253). In addition, if there are such a thing as political truths at all, then one could argue that it is harder to find them if one is just offered one perspective or view (which might be wrong), or—for example in the spirit of James or Dewey—that truth is the outcome of a process and that democracy needs pluralism (for more on pragmatism and pluralism see for example [11] and [19]). Epistemic bubbles are a hindrance to any form of democracy based on fallibilist and pluralist political epistemologies.

But the phenomenon of epistemic bubbles is also problematic with regard to *epistemic agency*. First, in this case algorithms control my epistemic environment, not me. While selection of information is always necessary, here it is not me who does the selection but an algorithm (and perhaps others, humans, who use that algorithm). Second, if I am not exposed to different views, the quality of my belief formation is low: I might have missed a better view but did not know it existed and I am not exposed to opposing views that could help me to reflect on my existing beliefs. It also becomes unlikely and harder to revise my beliefs, given that they are constantly being confirmed by others in my bubble. They are neither discussed nor contested. This is anti-democratic according to deliberative and agonistic models and may again support authoritarian and totalitarian tendencies. If I am reduced to a mere mouthpiece of my bubble, I cannot be a political agent in any (strong or “thick”) democratic sense. On the contrary, I am ready to be the extension of an authoritarian or totalitarian regime, in which interest it is that I become, am, and remain part of the epistemic bubble it creates or supports. I am more a political patient than a political agent. While usually epistemic bubbles are not intended, they can be used for totalitarian purposes or at least contribute to creating the conditions for totalitarianism. In so far as the use of AI helps to create epistemic bubbles, it is thus a danger for both epistemic and political agency in a democracy.

Again the “if” and the “in so far” qualifications are important here: the argument relies on the assumption that epistemic bubbles are created and effective. For example, Dubois and Blank [18] have argued that current media environments also offer a lot of choice and that this allows individuals interested in politics to consume a wide variety of media, which moderates the impact of echo chambers. Others have observed depolarization tendencies (Beam et al. [5]). But in so far as there *is* an epistemic bubble effect and polarization related to AI, I have offered an argument for why this might be problematic for democracy, based on the concept of epistemic agency.

Another way citizens’ epistemic agency may be influenced in non-intended ways is the very nature of the knowledge offered by AI and data science, statistical knowledge, and the way it tends to be defaulted in political situations. The issue is not statistical or mathematical knowledge as such, but the way it becomes a default in AI-assisted political contexts as opposed to scientific knowledge about causal relations. When AI—or more precisely, people using AI—offer us statistics in an easy way, directly or in the form of categorization or recommendation, we are less likely to look for causal relations. Finding scientific knowledge often requires some work, whereas today statistical knowledge is served to us abundantly in the form of a search result or a recommendation, enabled by an AI algorithm, or in news that reaches us via social media. For example, if I search for more information about the COVID-19 pandemic, an algorithm used by a search engine or a digital social media platform might lead me to conspiracy theories based on a statistical correlation between people who search for this topic and people who believe in conspiracy theories. In addition, due to the popularity of data and data analysis, the government might bombard me with statistical information about COVID-19. But causal evidence concerning what is happening is less likely to surface, and we are not encouraged to find out. Epistemic agency, it seems, is hardly needed when we have recommendations and numbers.

To understand why this can be a problem for democratically relevant epistemic agency, consider the following though experiment initially offered by Bondy [8], which is offered in another context (a mainstream epistemology discussion about voluntarism) but which I adapt for my purposes to include the use of AI. Bondy gives the example of Claire, who was raised in white supremacist environment and comes to believe that people with a white skin color (her skin color) are superior to others, but later learns that there is no scientific evidence for her belief. Will she be able to exercise epistemic agency and drop the belief or at least no longer use it in her future deliberations? The question I add is now: Will Claire be able to exercise that kind of epistemic agency (belief revision) if she is offered statistical knowledge provided by AI that shows, in spite of there being no causal evidence for it, that white people tend to be more successful in her society? I suspect that the statistical knowledge will play an important role in preventing her to exercise her epistemic agency. While there is no technological determinism here (Claire is still free to revise her beliefs), the defaulting of the knowledge that correlates skin color with success at least makes it *more difficult* for her to engage in belief revision with regard to that particular belief. Furthermore, the recommender and search algorithms she uses will likely lead her to information from (other) white supremacists, which again does not encourage her to exercise her epistemic agency. Getting such recommendations and getting such information from “friends” is easy; finding the truth is a lot harder. Even without intended manipulation of her beliefs, there is likely to be an unintended effect on Claire’s epistemic agency.

This is a thought experiment but can easily be turned into a real-world example about white supremacists in the US, who are unlikely to change their beliefs if they are insufficiently exposed to other views (if they are in an epistemic bubble) and to knowledge about causal relations, and if they operate in an environment in which statistical knowledge about society, rather than scientific knowledge, fuels the search engines and recommender systems they use. Far right movements such as QAnon, who reject scientific expertise, benefit from these effects. The far right does not need its followers to exercise epistemic agency.

My concern here is not that statistics lies (this problem is not about truth, because we can assume here that the statistical knowledge is true), or that its knowledge can easily be misused in projects that aim at manipulation (already before AI, statistics has been used to mislead and manipulate people), but rather that helped by AI, statistics tends to seduce people into focusing on correlations and not doing the more time-consuming work of checking the scientific evidence about causal relations. Defaulting statistical knowledge, AI thus offers an “easy” epistemic route via correlations, which does not require much effort on the part of citizens as epistemic agents, thereby hiding a more complex reality. Again, the correlation can be true. But nevertheless, it is not all that there is to say and as Claire’s example shows, on itself it might be misleading. Just as in the case of epistemic bubbles, there is a reduction of the knowledge space. Unless one is educated to open up that space and unless measures are taken to actively address the problem, this leaves citizens in the hands of anyone who wants to make use of this epistemic phenomenon to push through a particular view based on the correlation detected by AI. Citizens without training in statistics (most citizens) are especially vulnerable to this, since they are likely to be unaware of the limitations of statistical knowledge. A technocratic government can make use of this low epistemic power on the part of citizens to gain and maintain power. It can argue that given citizens’ ignorance, it is unsafe to have them in charge; it is better to give power to the experts and those who use them for their political purposes. While it could already do this by using statistics without AI, the use of AI makes this kind of knowledge more default and in that way, it may support technocracy. For example, the statistical knowledge comes to us in the forms of recommendations or categorizations. People will use this kind of knowledge as a default; only experts know what is going on or have easy access to other types of knowledge.

Normatively speaking, such threats to epistemic agency are also threats to the autonomy of citizens. Consider the following comparison. Cases where non-intended effects such as epistemic bubbles and the defaulting of statistical correlational information are used on purpose can be helpfully compared with nudging [53]. The idea of nudging is to influence the choices of people by changing their choice architecture, without constraining what Berlin [7] calls their negative freedom, their freedom from constraint: people are still free to choose. However, their choice architecture and environment are structured in specific ways on purpose, to influence their choices. For example, a supermarket may put snacks near the checkout in order to stimulate sales of snacks. But in the spirit of Sunstein and Thaler it can also be used for good purposes, in this case to present healthy food. However, it is questionable if it respects the freedom of citizens and consumers: Thaler and Sunstein are right to say that people are externally and negatively free, in the sense that no-one is directly interfering with their freedom of choice; however, people’s internal autonomy and agency seems to be compromised since they are influenced in subconscious ways.

Similarly, influencing belief formation and belief revision by using AI changes the epistemic architecture and the epistemic environment of people. This does not render them unfree when it comes to changing their beliefs. Nobody forces them to change or keep their beliefs. Claire is free to adopt non-racist beliefs. However, citizens’ belief formation and belief revision processes are manipulated by changing the epistemic architecture and environment in such a way that it becomes *more difficult* for them to do this. Claire has all the negative freedom in the world with regard to changing her beliefs since nobody constrains her or forces her with regard to her beliefs. For example, nobody forces her to keep her white supremacist beliefs. But her epistemic agency and autonomy is compromised by the intended or non-intended influence that are created by the AI-enabled and social media-supported epistemic bubbles and defaulting of statistical knowledge. This is not only problematic because it leads to the persistence of particular beliefs in society (in this case racism and white supremacism); it is also a threat to Claire’s epistemic agency and epistemic autonomy, which in turn diminishes her political agency in a democratic society. Today citizens in democracies risk to find themselves in similar situations, often without knowing that the intended or non-intended influencing takes place.

Note that this analysis in terms of epistemic and political agency can also be connected to other political concepts and principles. For example, the phenomena described here could also be analyzed in terms of what Dotson [17] calls ‘epistemic oppression’, which concerns ‘infringement on the ability to utilize persuasively shared epistemic resources’ for knowledge production (p. 116). In addition, one could not only make links to the principles of freedom and autonomy but also to questions regarding justice and fairness, discrimination, bias, and so on. For example, one could use the bridge concept of ‘epistemic justice’ (see for example [20] or [44]) and related concepts such as ‘epistemic network injustice’ which concerns connections to like-minded people [50]: the opposite of epistemic bubbles, a total lack of what Spiekermann calls ‘epistemic solidarity’ (p. 98), is also politically problematic. Epistemic forms of exclusion and injustice are of course politically relevant and impact democracy. However, here I limit my discussion to epistemic agency and the specific political risks for democracy described, while acknowledging that the present discussion could be further developed by connecting it to a wider ecology of political–philosophical concepts.

## Conclusion with policy recommendations

In this paper, I have given an overview of a number of ways in which the knowledge base of democracy risks to become eroded by the use of AI. This includes fake news and misinformation, but also other phenomena such as epistemic bubbles and the defaulting of statistical information. These phenomena may be problematic in all kinds of (other) ways, but I have focused on their impact on *epistemic agency*, which in turn threatens political agency in a democracy. While acknowledging that the empirical reality is more complex and that epistemic agency has already been under attack before AI came to the scene, I have identified some lines of philosophical argument that can be pursued to support concerns about these phenomena in relation to democracy.

To the extent that these phenomena are a reality and if these arguments hold, then if we care about democracy, it is highly recommended to take measures to avoid, limit, or reduce these problems and protect or even enhance epistemic and political agency in the light of AI and related technological-social phenomena. For example, measures could be taken to render manipulation less likely and to boost the epistemic agency of citizens by means of education, and the use and development of AI and data science could be regulated in such a way that the risks described here are avoided or at least mitigated.

For example, educational programs should include lessons about statistics and its limitations, and should encourage citizens to cultivate their reasoning capacities, to ‘experience epistemic doubt, think critically, and understand diverse views’, as Paakkari and Sørensen [41] recommend in the context of dealing with epistemic bubbles in the context of the pandemic. Similarly, one could help people to develop epistemic virtues as responsible epistemic agents, as Gunn and Lynch [26] propose when observing increased epistemic arrogance and discounting the credibility of others on the Internet. Those who think that they know everything already not only lack epistemic virtue but have in fact a low degree of epistemic agency.

But there is also a role for developers of AI and the companies and organizations they work for. The AI algorithms used by social media platforms could be changed in such a way that they enable disruption of epistemic bubbles. Bozdag and van den Hoven [10] offer some examples of software designs that try to ‘break’ filter bubbles, while noting that such tools are often limited in terms of the range of democracy models they use. This is a more general problem with many technical tools for democracy; here (political) philosophy can play a role [48]. We need more research that links democracy theory to technical work. Policy makers need to offer a framework that fosters such interdisciplinary research and that encourages and requires AI developers and their companies to develop democracy-proof AI.

I write “requires” since given the role of Big Tech and more generally private corporations, developing more politically responsible AI is not just a matter of appealing to the individual responsibility of AI researchers, but also necessitates policies that push companies to invest in, create, and employ AI that is good for democracy. Self-regulation is unlikely to succeed when companies make profit from some of the effects discussed in this paper. Regulation and a new distribution of power is needed. In a democracy, the future of AI and society should be decided by the people, not by a handful of companies and their leaders.

Furthermore, a framework for the democratic governance of AI (a need for which is increasingly recognized, see for example [6] for a European context) may need new legal concepts. For example, Risse [47] has proposed epistemic rights as fourth generation human rights in order to protect what he calls ‘epistemic actorhood’ (p. 351).; one could demand the same to protect epistemic and political agency. For example, based on the analysis offered here, rights to education could be extended to, or at least interpreted as, including the right to receive training in reflecting on one’s beliefs and discuss them with others in AI-pervaded online environments, in considering and confronting the views of others under such circumstances, and in dealing with AI-generated information and recommendations based on statistical information.

Finally, given that AI development and its political-epistemic influence has global reach, we do not only need national frameworks but also global governance of AI. Here, a major hurdle is that we lack global political institutions.  In contrast to the powerful Big Tech corporations that decide our technological future, our current institutions are insufficiently supranational.

My sense is that we are only at the very beginning of understanding the social-epistemic phenomena created by AI and other digital technologies. More research is needed to further understand and evaluate what is going on and what it means for democracy in the twenty-first century. This paper, which was limited to offering conceptual arguments and justifications based on epistemic agency, is only one example of how political epistemology can be done in a way that is responsive to technological–social transformations: transformations that emerged only recently but that most likely will continue to significantly change our epistemic and political world.

## Footnotes

1 https://www.nytimes.com/2020/06/27/technology/pizzagate-justin-bieber-qanon-tiktok.html.

2 https://www.brookings.edu/techstream/how-china-uses-search-engines-to-spread-propaganda/.

3 https://www.wired.com/story/opinion-authoritarian-regimes-could-exploit-cries-of-deepfake/.

4 https://www.theguardian.com/technology/ng-interactive/2019/jun/22/the-rise-of-the-deepfake-and-the-threat-to-democracy.

5 https://www.politico.eu/newsletter/ai-decoded/politico-ai-decoded-how-cambridge-analytica-used-ai-no-google-didnt-call-for-a-ban-on-face-recognition-restricting-ai-exports/.
